# One-Year Outcomes and Trends over Two Eras of Transcatheter Aortic Valve Implantation in Real-World Practice

**DOI:** 10.3390/jcm11051164

**Published:** 2022-02-22

**Authors:** Giuliano Costa, Paola D’Errigo, Stefano Rosato, Fausto Biancari, Andrea Marcellusi, Giuseppe Tarantini, Gennaro Santoro, Massimo Baiocchi, Diego Maffeo, Claudia Fiorina, Francesco Cerza, Giovanni Baglio, Tatu Juvonen, Gabriella Badoni, Roberto Valvo, Fulvia Seccareccia, Marco Barbanti, Corrado Tamburino, on behalf of the OBSERVANT II Research Group

**Affiliations:** 1 Division of Cardiology, A.O.U. Policlinico “G. Rodolico-San Marco”, University of Catania, 95123 Catania, Italy; giulianocosta90@gmail.com (G.C.); roberto_valvo@hotmail.it (R.V.); tambucor@unict.it (C.T.); 2 National Centre for Global Health, Istituto Superiore di Sanità, 00161 Rome, Italy; paola.derrigo@iss.it (P.D.); stefano.rosato@iss.it (S.R.); gabriella.badoni@iss.it (G.B.); fulvia.seccareccia@iss.it (F.S.); 3 Heart and Lung Center, Helsinki University Hospital, University of Helsinki, 00029 Helsinki, Finland; faustobiancari@yahoo.it (F.B.); tatu.juvonen@oulu.fi (T.J.); 4 Research Unit of Surgery, Anesthesiology and Critical Care, University of Oulu, 90570 Oulu, Finland; 5 Centre for Economic Evaluation and HTA (EEHTA), Centre for Economic and International Studies (CEIS), Faculty of Economics, University of Rome “Tor Vergata”, 00133 Rome, Italy; andrea.marcellusi@gmail.com; 6 Division of Cardiology, Department of Cardiac, Thoracic and Vascular Sciences, University of Padova, 35122 Padova, Italy; giuseppe.tarantini.1@gmail.com; 7 Division of Diagnostic and Interventional Cardiology, Fondazione “G. Monasterio” CNR—Tuscany Region for the Medical Research and Public Health, 94100 Massa, Italy; santorogenn@gmail.com; 8 Division of Cardiology, Department of Cardiac, Thoracic and Vascular Diseases, Policlinico Sant’Orsola, 40138 Bologna, Italy; dr.massimo.baiocchi@gmail.com; 9 Interventional Cardiology Unit, Cardiovascular Department, Fondazione Poliambulanza, 25124 Brescia, Italy; diego.maffeo@poliambulanza.it; 10 Division of Cardiology, Cardiothoracic Department, Spedali Civili, 25123 Brescia, Italy; clafiorina@yahoo.it; 11 Department of Epidemiology, Italian National Agency for Regional Healthcare Services, 00147 Rome, Italy; cerza@agenas.it (F.C.); baglio@agenas.it (G.B.)

**Keywords:** transcatheter aortic valve implantation, outcomes, trends, OBSERVANT

## Abstract

Background: Data reflecting the benefit of procedural improvements in real-world transcatheter aortic valve implantation (TAVI) practice are sparse. Aims: To compare outcomes and trends of two TAVI eras from real Italian practice. Methods: A total of 1811 and 2939 TAVI patients enrolled in the national, prospective OBSERVANT and OBSERVANT II studies in 2010–2012 and 2016–2018, respectively, were compared in a cohort study. Outcomes were adjusted using inverse propensity of treatment weighting and propensity score matching. Results: The median age (83.0 (79.0–86.0) vs. 83.0 (79.0–86.0)) and EuroSCORE II (5.2 (3.2–7.7) vs. 5.1 (3.1–8.1)) of OBSERVANT and OBSERVANT II patients were similar. At 1 year, patients of the OBSERVANT II study had a significantly lower risk of all-cause death (10.6% vs. 16.3%, Hazard Ratio (HR) 0.63 (95% Confidence Interval (CI) 0.52–0.76)) and rehospitalization for heart failure (HF) (14.3% vs. 19.5%, Sub-distribution HR 0.71 (95%CI 0.60–0.84)), whereas rates of stroke (3.1% vs. 3.6%) and permanent pacemaker implantation (PPI) (16.6% vs. 18.0%) were comparable between study groups. Conclusions: Age and risk profile among patients undergoing TAVI in Italy remained substantially unchanged between the 2010–2012 and 2016–2018 time periods. After adjustment, patients undergoing TAVI in the most recent era had lower risk of all-cause death and rehospitalization for HF at 1 year, whereas rates of stroke and PPI did not differ significantly.

## 1. Introduction

Transcatheter aortic valve implantation (TAVI) has rapidly expanded its indications over the last decade, supported by the excellent results of randomized clinical trials (RCTs). Indeed, this therapy evolved from treating elder, high-risk patients to younger, low-risk patients, based on improvements in outcomes ensured by advances in the field [1,2,3,4]. However, a notable impact of these improvements has been demonstrated by the reduction in risk profile of the population, thus making it challenging to isolate the real effect of new TAVI devices and procedural optimization [5,6]. In this context, it appears useful to investigate trends and clinical outcomes of daily TAVI practice over recent years. Therefore, the aim of this analysis was to assess differences in population and procedural characteristics of TAVI and to compare clinical outcomes between patients enrolled in the Observational Study of Effectiveness of AVR-TAVI procedures for severe Aortic steNosis Treatment (OBSERVANT) and Observational Study of Effectiveness of TAVI with new generation deVices for severe Aortic stenosis Treatment (OBSERVANT II) studies, which substantially reflect the early and expansion TAVI eras in Italy, respectively.

## 2. Materials and Methods

Data for the present analysis were obtained from the OBSERVANT and OBSERVANT II datasets. OBSERVANT was a national observational, prospective, multicenter cohort study that enrolled consecutive AS patients who underwent TAVI or surgical aortic valve replacement (SAVR) at 93 Italian centers (34 cardiology centers and 59 cardiac surgery centers) between December 2010 and June 2012 [7].

OBSERVANT II was a national observational, prospective, multicenter cohort study that enrolled consecutive AS patients who underwent TAVI at 30 Italian centers of cardiology between December 2016 and September 2018 [8]. Only 28 centers met the minimum data quality criteria required by the study protocol and their data are included in this analysis [8]. The Ethical Committee of each participating centers granted the permission to participate in the OBSERVANT and OBSERVANT II studies. All patients included in these studies gave informed consent to the scientific treatment of their data on an anonymous form. Data on baseline characteristics, operative details and adverse events occurred during the index hospitalization were prospectively collected into an electronic case report form [8]. Data on adverse events occurred after hospital discharge were gathered by a linkage with the National Hospital Discharged Records database provided by the Italian Ministry of Health and other administrative databases available through a collaboration with the Italian National Program for Outcome Evaluation (PNE-AGENAS). Linking to these national registries guaranteed complete follow-up data on outcomes at 1-year follow-up.

**Figure 1:**
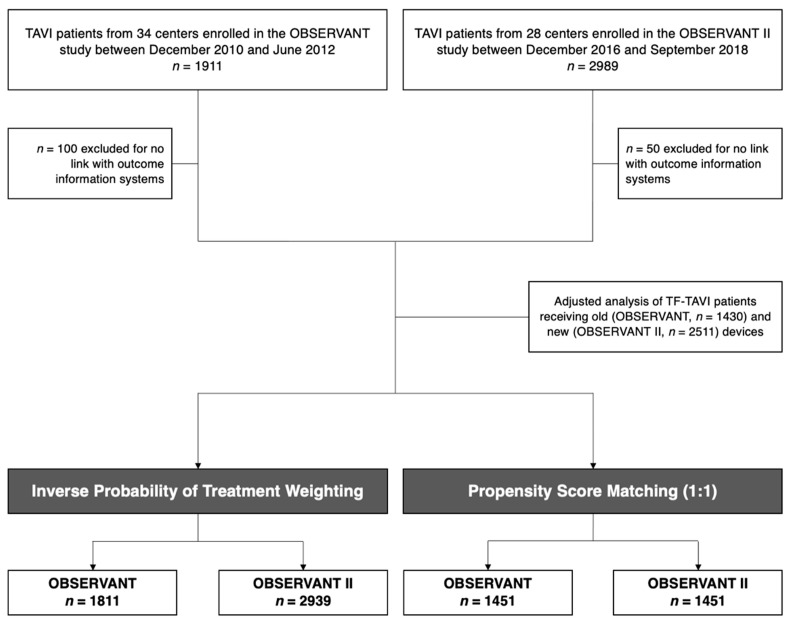
Study participants flowchart.

For the purposes of this analysis, we considered all patients undergoing TAVI and enrolled in the OBSERVANT and OBSERVANT II studies (Figure 1). Changes in baseline, procedural characteristics and post-procedural care were analyzed comparing the crude overall populations of the two studies. Procedural and clinical outcomes were adjusted by taking into account the baseline characteristics of the two populations.

Longitudinal changes in patients’ and procedural characteristics, and post-procedural care between the time periods of the two studies were assessed. Primary clinical endpoints were all-cause death, stroke, and hospital readmission due to heart failure (HF) at 1 year. Secondary clinical outcomes of interest were myocardial infarction (MI) and permanent pacemaker implantation (PPI) at 1 year, and adverse events occurring during the index hospitalization.

Inverse probability of treatment weighting (IPTW) based on propensity score (PS) was used as the primary tool to adjust for baseline confounding variables between the compared groups. One-to-one PS matching (PSM) with the nearest neighbor method was used as a sensitivity analysis. Details of the statistical analysis are reported in the Supplementary Materials.

## 3. Results

This analysis included a total of 1811 patients enrolled in OBSERVANT from December 2010 to June 2012 and 2939 patients enrolled in the OBSERVANT II registry from December 2016 to September 2018 (Figure 1). The median age (83.0 (79.0–86.0) vs. 83.0 (79.0–86.0); *p* = 0.89) and EuroSCORE II (5.2 (3.2–7.7) vs. 5.1 (3.1–8.1); *p* = 0.26) of OBSERVANT and OBSERVANT II patients were similar.

Patients enrolled in the OBSERVANT study were more frequently female (58.8% vs. 54.8%) with higher prevalence of chronic obstructive pulmonary disease (COPD) (28.0% vs. 16.0%), renal failure (12.1% vs. 10.3%), coronary artery disease (30.6% vs. 25.6%), peripheral artery disease (PAD) (26.3% vs. 18.9%), and critical preoperative state (4.3% vs. 2.6%). Patients enrolled in the OBSERVANT II study had a higher body mass index (BMI) (25.8 (23.2–29.1) vs. 25.5 (22.9–28.3)) and were more frequently in NYHA class III or IV (72.0% vs. 66.5%), with a higher rate of moderate or severe mitral regurgitation (31.9% vs. 29.0%).

OBSERVANT II patients underwent TAVI more frequently through a transfemoral approach (90.8% vs. 82.2%), less frequently under general anesthesia (17.7% vs. 35.4%), and more frequently underwent concomitant percutaneous coronary intervention (5.1% vs. 3.2%). In-hospital length of stay was significantly lower for OBSERVANT II patients (9 (6–14) days vs. 10 (8–16) days) with shorter post-procedural stay in the intensive care unit (1 (0–2) day vs. 2 (1–3) days).

**Figure 2:**
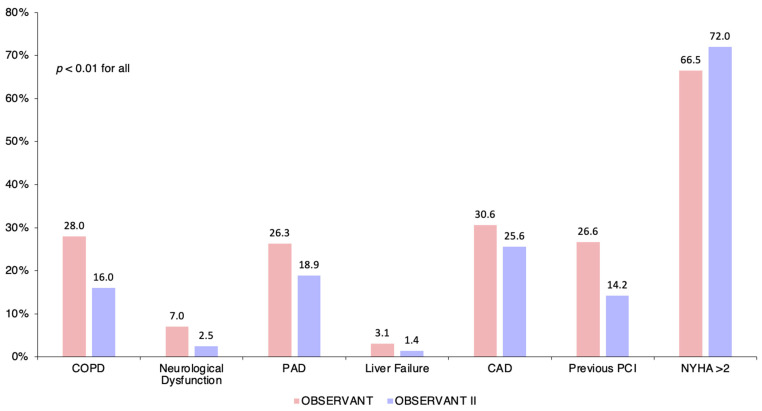
Crude baseline differences of patients undergoing transcatheter aortic valve implantation and enrolled in the OBSERVANT and OBSERVANT II studies. CAD, coronary artery disease; COPD, chronic obstructive pulmonary disease; NYHA, New York Heart Association; PAD, peripheral artery disease; PCI, percutaneous coronary intervention.

**Figure 3:**
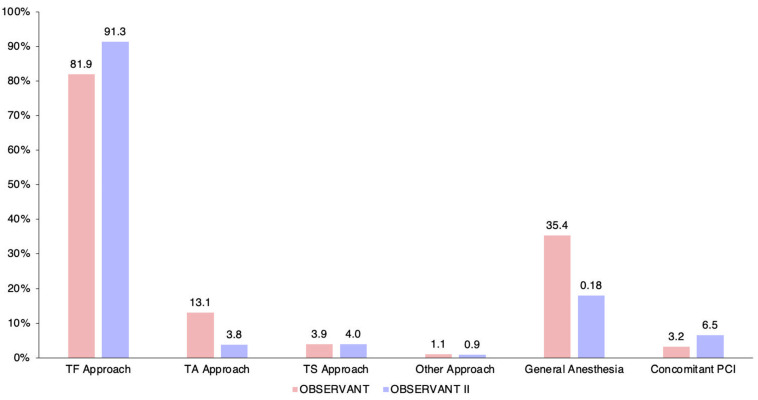
Crude procedural differences of patients undergoing transcatheter aortic valve implantation and enrolled in the OBSERVANT and OBSERVANT II studies. PCI, percutaneous coronary intervention; TA, trans-apical; TF, trans-femoral; TS, trans-subclavian.

Patient and procedural characteristics of the OBSERVANT and OBSERVANT II cohorts are summarized in Table 1 and Figure 2, and in Figure 3 and Supplementary Table S1, respectively.

During the index hospitalization, patients of the OBSERVANT II study had lower rates of acute kidney injury (AKI) (1.4% vs. 6.6%), vascular complications (2.4% vs. 6.3%) and MI (0.3% vs. 0.9%), whereas similar rates of PPI (13.2% vs. 14.9%) and stroke (0.6% vs. 1.1%) were reported between OBSERVANT II and OBSERVANT patients.

**Figure 4:**
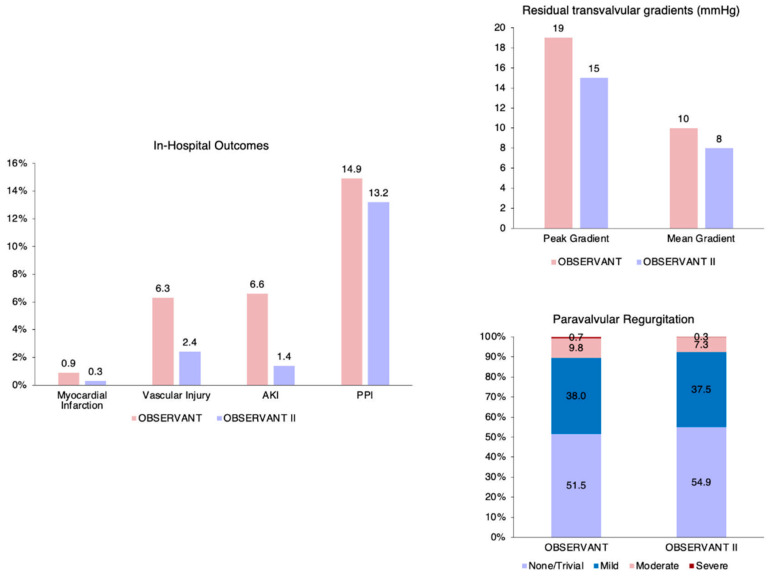
In-hospital clinical and echocardiographic outcomes after adjustment. AKI, acute kidney disease; PPI, permanent pacemaker implantation.

Patients enrolled in the OBSERVANT II study had lower values of residual transvalvular gradients (mean gradient 8 (5–11) mmHg vs. 10 (7–12) mmHg) and moderate or severe paravalvular regurgitation (PVR) (7.6% vs. 10.5%) at pre-discharge echocardiographic assessment. Adjusted in-hospital outcomes are reported in Figure 4 and Supplementary Table S2.

**Figure 5:**
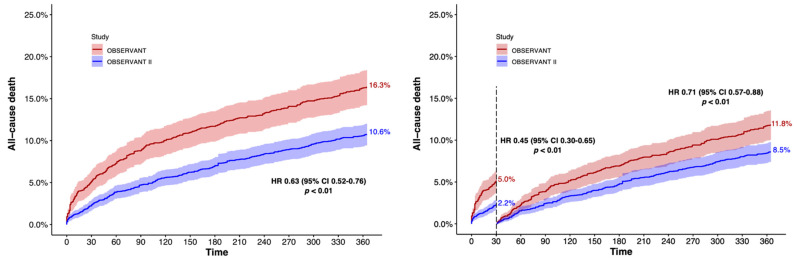
Adjusted 1-year Kaplan–Meier survival curve and landmark analysis for all-cause death. CI, confidence interval; HR, hazard ratio.

Patients of the OBSERVANT II study had a significantly lower risk of all-cause death (10.6% vs. 16.3%, Hazard ratio (HR) 0.63 (95% Confidence Interval (CI) 0.52–0.76); *p* < 0.01) at 1 year compared to patients enrolled in the OBSERVANT study (Table 2 and Figure 5). Landmark analysis showed that the benefit in terms of all-cause death for patients enrolled in the OBSERVANT II study was greater within the first 30 days after the procedure (2.2% vs. 5.0%, HR 0.45 (95% CI 0.30–0.65); *p* < 0.01), and maintained thereafter (8.5% vs. 11.8% from 30 days to 1 year, HR 0.71 (95% CI 0.57–0.88); *p* < 0.01) (Supplementary Table S3 and Figure 5).

**Figure 6:**
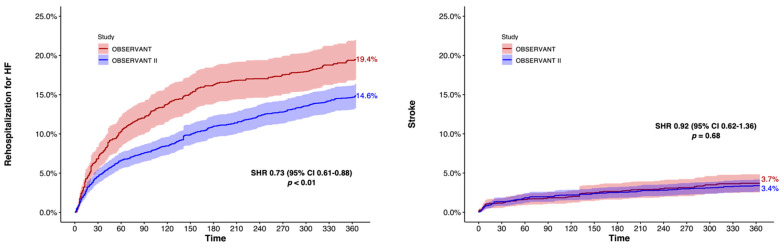
Adjusted 1-year Fine and Gray cumulative incidence analysis for rehospitalization for heart failure and stroke. CI, confidence interval; SHR, sub-distribution hazard ratio.

The risk of rehospitalization for HF (14.3% vs. 19.5%, HR 0.71 (95% CI 0.60–0.84); *p* < 0.01) was lower for OBSERVANT II patients, whereas rates of stroke (3.1% vs. 3.6%, HR 0.85 (95% CI 0.60–1.21); *p* = 0.14), PPI (16.6% vs. 18.0%, HR 0.92 (95% CI 0.78–1.09); *p* = 0.11) and MI (1.7% vs. 2.0%, HR 0.87 (95% CI 0.55–1.35); *p* = 0.34) were comparable between patients of the OBSERVANT II and OBSERVANT studies at 1 year (Table 2 and Figure 6).

## 4. Discussion

Although large RCTs have rapidly driven TAVI from treatment of high-risk patients to its adoption as an alternative to surgery even for low-risk patients over the past decade, the impact of advancement in devices and of procedural optimization in real-world practice has been poorly investigated. This study aimed to analyze changes in TAVI practice and its clinical outcomes, comparing patients enrolled in the OBSERVANT II study between 2016 and 2018, and patients previously enrolled in the OBSERVANT study between 2010 and 2012.

The main findings were: (1) patients’ age and predicted risk did not differ between the two study periods, but OBSERVANT II patients had less comorbidities; (2) in the OBSERVANT II period, patients underwent TAVI more frequently through a transfemoral approach under local anesthesia, had fewer complications and their hospitalization period was shorter; (3) patients undergoing TAVI between 2016 and 2018 showed a lower risk of all-cause death and hospitalization for HF at 1 year compared to patients treated between 2010 and 2012, whereas rates of stroke, MI and PPI were similar.

National TAVI registries revealed a downward trend in patients’ age and predicted risk during the past decade worldwide, based on the evidence of large trials [5,6]. Differently, our comparison did not confirm this tendency in Italy, where TAVI patients had similar age and predicted risk scores between the 2010–2012 and 2016–2018 periods. This finding requires specific comments: first, patients undergoing TAVI during the first time period took into account had more comorbidities that are not considered within the algorithms of the most used risk prediction tools; second, the median risk score in the first OBSERVANT study was remarkably lower than that reported in other national registries during the same period, thus suggesting that, in Italy, a shift toward a reduction in risk profile of TAVI candidates occurred a few years before. Additionally, this is probably the result of the different timing in diagnosis of severe aortic stenosis in Italy, which continues to be detected in the elderly when symptoms are already at an advanced stage. This is confirmed by the higher rate of patients with advanced functional NYHA class in the more recent time period of the OBSERVANT II study.

Besides the changes in TAVI patient characteristics, over the past decade, we have witnessed the optimization of the TAVI procedure and of the care pathways, which have been shown to impact early and mid-term outcomes [9,10,11]. Indeed, advancements in TAVI devices, a better pre-procedural assessment due to the widespread adoption of ECG-gated computed tomography angiography (CTA), as well as optimization in post-procedural care, largely showed improvements in procedural outcomes and allowed a more rapid discharge with a lower utilization of hospital resources. Patients enrolled in the OBSERVANT II study between 2016 and 2018 received new generation devices and underwent TAVI more frequently through a transfemoral approach under local anesthesia. Confirming the data of recent large TAVI registries that have investigated the benefit of a minimalistic approach [12,13], they showed lower rates of in-hospital complications and a shorter length of stay, with a lower need of stay in intensive care units after the procedure. As a result of the aforementioned changes in TAVI practice over the past decade, different large nationwide studies showed significant improvements in TAVI outcomes. A temporal trends analysis comparing patients enrolled in the FRANCE 2 (French Aortic National CoreValve and Edwards 2) and in the FRANCE TAVI (French Transcatheter Aortic Valve Implantation) registries between January 2010 and January 2012 and from January 2013 to December 2015, respectively, showed that in-hospital and 30-day mortality rates after TAVI significantly decreased from 8.2% and 10.1% in FRANCE 2 to 4.4% and 5.4% in the FRANCE TAVI study, respectively [5]. Differently, stroke and potentially life-threatening complications, such as annulus rupture or aortic dissection, remained stable over the time periods of the two registries. The STS-ACC TVT Registry (Society of Thoracic Surgeons–American College of Cardiology Transcatheter Valve Therapy Registry) collected data of 276,316 patients undergoing TAVI at sites in all U.S. states from 2011 to 2019 [6]. Over this time period, annual TAVI volume increased every year, extending to over 8000 low-risk patients in 2019. The 30-day all-cause mortality rate has stepwise decreased from 7.2% to 2.5%, whereas stroke has showed a slower decrease and PPI has remained unchanged. At 1 year, all-cause death decreased from 26.4% in 2012 to 13.7% in 2017, and is expected to further reduce given the treatment of lower-risk patients during recent years. Similarly, we reported a marked reduction in the risk of 1-year all-cause death and rehospitalization for HF of patients enrolled in the OBSERVANT II patients in the 2016–2018 period compared to patients enrolled in the OBSERVANT study between 2010 and 2012. Furthermore, the rates of stroke, PPI and MI after TAVI did not differ between TAVI practice over the two registries. The goodness of adjustment was corroborated by the correspondence of results using PS Matching as sensitive analysis and by the standardized mean difference of baseline variables lower than 12%. These results were substantially confirmed in the sub-analysis of TF-TAVI patients, meaning that the improvements are mostly related to the advancements in device design and operator’s experience, rather than the shift itself to a less invasive approach. Nevertheless, it has to be remarked that in an exploratory analysis, the benefit in mortality was more remarkable in patients treated with different generations of the self-expanding CoreValve/Evolut TAVs family (Supplementary Materials). The reasons behind this finding are mostly: (1) the widespread adoption of preprocedural CTA assessment for all TAVI recipients during the last few years of Italian practice and (2) the numerous improvements introduced with Evolut R/PRO iterations, including the recapturability. The use of balloon-expandable SAPIEN family devices was more restricted, especially during the early Italian TAVI experience, at least in part due to the need of a more careful sizing evaluation (either by pre-procedural CTA or transesophageal echocardiogram), but their more predictable deployment ensured better procedural outcomes and device success rates. Contrarily, first generation self-expanding CoreValve TAV had been widely used during the first era of Italian TAVI practice, making it possible to achieve procedural success even with the use of only angiographic guidance (i.e., for patients with severe renal failure who could not undergo CTA assessment). New generation self-expanding Evolut R/PRO devices have brought about a more predictable deployment and the feature of recapturing/repositioning the device during implantation if needed. These new characteristics, together with the widespread adoption of pre-procedural CTA in clinical practice (that can now be used with specific protocols minimizing the usage of contrast dye in patients with renal failure) led to a significant improvement in procedural outcomes. Nevertheless, the absence of data about pre-procedural assessment in both the OBSERVANT and OBSERVANT II registries as well as of TAV sizing details does not allow us to confirm the aforementioned assumptions.

### Limitations

The present study has several limitations which should be acknowledged. First, this is a comparative analysis of patients who underwent TAVI included in two national prospective studies. Despite the goodness of adjustment, we cannot exclude confounding bias related to the lack of randomization. Second, the OBSERVANT II study involved a larger variety of TAV devices that might have had different rates of moderate or severe paravalvular regurgitation, which in turn has shown to impact on mortality. Analysis of the impact of each TAV device on early and intermediate outcomes was not performed because it was out of the scope of the present analysis. Third, the OBSERVANT studies did not collect data on cardiovascular mortality. Finally, the absence of data regarding pre-procedural assessment in each center did not allow us to recognize in which measure the change in clinical practice over the time periods of the two studies affected the results of our analysis.

## 5. Conclusions

Age and risk profile among patients undergoing TAVI in Italy remained substantially unchanged between the 2010–2012 and 2016–2018 time periods. A shift towards to a higher adoption of the transfemoral approach has been detected. Patients undergoing TAVI in the era of new generation devices and optimized pre-procedural assessment showed better procedural outcomes, shorter in-hospital stay, and lower risk of all-cause death and rehospitalization for HF at 1 year. Rates of stroke, PPI and MI did not differ compared to patients treated during the early TAVI experience.

## Figures and Tables

**Table 1:** Baseline characteristics of population before and after baseline covariates adjustment.

	Before Adjustment			IPTW Adjustment			PSM Adjustment		
	OBS (*n* = 1811)	OBS II (*n* = 2939)	*p* Value	OBS (*n* = 1811)	OBS II (*n* = 2939)	*p* Value	OBS (*n* = 1451)	OBS II (*n* = 1451)	*p* Value
Age	83.0 (79.0–86)	83.0 (79.0–86.0)	0.892	83.0 (78.0–86.0)	83.0 (79.0–86.0)	0.429	83.0 (79.0–86.0)	83.0 (79.0–86.0)	0.941
Female sex	1064 (58.8)	1611 (54.8)	<0.01	1021 (56.4)	1652 (56.2)	0.921	836 (57.6)	824 (56.8)	0.680
EuroSCORE II	5.2 (3.3–7.7)	5.1 (3.1–8.1)	0.259	5.0 (3.0–7.3)	5.3 (3.2–8.3)	0.106	4.9 (3.1–7.3)	5.4 (3.2–8.4)	0.050
BMI	25.5 (22.9–28.3)	25.8 (23.2–29.1)	<0.01	25.7 (22.9–28.4)	25.7 (23.2–28.9)	0.435	25.7 (22.9–28.4)	25.4 (22.9–28.4)	0.587
GSS 2 or 3	436 (24.1)	634 (21.6)	0.045	393 (21.7)	655 (22.3)	0.667	321 (22.1)	313 (21.6)	0.753
Diabetes	481 (26.6)	809 (27.5)	0.481	489 (27.0)	808 (27.5)	0.787	389 (26.8)	377 (26.0)	0.643
Severe renal failure	220 (12.1)	303 (10.3)	0.051	187 (10.3)	309 (10.5)	0.849	158 (10.9)	155 (10.7)	0.905
Dialysis	42 (2.3)	71 (2.4)	0.922	42 (2.3)	71 (2.4)	0.857	34 (2.3)	31 (2.1)	0.802
COPD	507 (28.0)	470 (16.0)	<0.01	359 (19.8)	591 (20.1)	0.780	337 (23.2)	312 (21.5)	0.285
Oxygen Therapy	108 (6.0)	96 (3.3)	<0.01	71 (3.9)	109 (3.7)	0.679	60 (4.1)	53 (3.7)	0.565
Neurological dysf.	126 (7.0)	74 (2.5)	<0.01	74 (4.1)	120 (4.1)	0.997	63 (4.3)	62 (4.3)	1.000
PAD	477 (26.3)	555 (18.9)	<0.01	398 (22.0)	647 (22.0)	0.970	349 (24.1)	349 (24.1)	1.000
Liver cirrhosis	56 (3.1)	42 (1.4)	<0.01	36 (2.0)	62 (2.1)	0.811	33 (2.3)	29 (2.0)	0.700
Active malignancy	70 (3.9)	125 (4.3)	0.547	74 (4.1)	118 (4.0)	0.929	57 (3.9)	54 (3.7)	0.847
PH	319 (17.6)	151 (5.1)	<0.01	181 (10.0)	270 (9.2)	0.473	136 (9.4)	129 (8.9)	0.699
Angina	49 (2.7)	138 (4.7)	<0.01	78 (4.3)	118 (4.0)	0.698	44 (3.0)	50 (3.4)	0.600
CAD	554 (30.6)	751 (25.6)	<0.01	505 (27.9)	802 (27.3)	0.720	420 (28.9)	414 (28.5)	0.838
Previous MI	315 (17.4)	434 (14.8)	0.017	273 (15.1)	464 (15.8)	0.526	236 (16.3)	228 (15.7)	0.723
Pre. aortic surgery	93 (5.1)	107 (3.6)	0.014	78 (4.3)	120 (4.1)	0.865	67 (4.6)	75 (5.2)	0.547
Previous PCI	482 (26.6)	416 (14.2)	<0.01	330 (18.2)	558 (19.0)	0.517	322 (22.2)	312 (21.5)	0.686
Previous CABG	228 (12.6)	299 (10.2)	0.012	194 (10.7)	320 (10.9)	0.850	168 (11.6)	177 (12.2)	0.646
AF	402 (22.2)	658 (22.4)	0.886	389 (21.5)	655 (22.3)	0.596	309 (21.3)	312 (21.5)	0.928
RBBB	111 (6.1)	201 (6.8)	0.366	129 (7.1)	200 (6.8)	0.773	98 (6.8)	95 (6.5)	0.882
NYHA > 2	1205 (66.5)	2116 (72.0)	<0.01	1262 (69.7)	2054 (69.9)	0.872	985 (67.9)	988 (68.1)	0.937
Critical status	78 (4.3)	77 (2.6)	<0.01	96 (5.3)	91 (3.1)	0.128	46 (3.2)	50 (3.4)	0.756
Hemoglobin (g/dL)	12.0 (10.0–13.0)	12.0 (11.0–13.0)	<0.01	12.0 (11.0–13.0)	12.0 (11.0–13.0)	0.685	12.0 (11.0–13.0)	12.0 (10.0–13.0)	0.959
**Echocardiographic parameters**									
LVEF	55.0 (45.0–60.0)	55.0 (48.0–60.0)	<0.01	55.0 (45.0–60.0)	55.0 (46.0–60.0)	0.963	55.0 (46.0–60.0)	55.0 (46.0–60.0)	0.545
Aortic mean gradient (mmHg)	48.0 (40.0–58.0)	46.0 (39.0–55.0)	<0.01	47.0 (40.0–56.0)	47.0 (40.0–55.0)	0.350	48.0 (40.0–57.0)	47.0 (40.0–56.0)	0.228
AVA (cm^2^)	0.7 (0.5–0.8)	0.7 (0.5–0.8)	<0.01	0.7 (0.5–0.8)	0.7 (0.5–0.8)	0.911	0.7 (0.5–0.8)	0.7 (0.5–0.8)	0.480
Grade 2 + MR	526 (29.0)	938 (31.9)	0.038	585 (32.3)	911 (31.0)	0.516	414 (28.5)	419 (28.9)	0.870

Continuous variables are reported as median and interquartile range (in parentheses). Categorical variables are reported as counts and percentages (in parentheses). Abbreviations: AF, atrial fibrillation; AVA, aortic valve area; BMI, body mass index; CABG, coronary artery bypass grafting; CAD, coronary artery disease; COPD, chronic obstructive pulmonary disease; GSS, Geriatric Status Scale; IPTW; inverse probability of treatment weighting; IQR, interquartile range; LVEF, left ventricular ejection fraction; MR, mitral regurgitation; NYHA, New York Heart Association; PAD, peripheral artery disease; PCI, percutaneous coronary intervention; PH, pulmonary hypertension; PSM, propensity score matching; RBBB, right bundle branch block.

**Table 2:** One-year outcomes after inverse probability of treatment weighting (IPTW) and propensity score matching (PSM) adjustment.

	OBS	OBS II	HR/SHR (95%CI)	*p*-Value
**IPTW adjustment**	**N = 1811**	**N = 2939**		
All-cause death	16.3%	10.6%	0.63 (0.52–0.76)	<0.001
Rehospitalization for HF	21.0%	14.9%	0.68 (0.58–0.80)	<0.001
Stroke	3.6%	3.1%	0.85 (0.60–1.21)	0.142
PPI	18.0%	16.6%	0.92 (0.78–1.09)	0.109
MI	2.0%	1.7%	0.87 (0.55–1.35)	0.341
**PSM adjustment**	**N = 1451**	**N = 1451**		
All-cause death	16.3%	11.2%	0.67 (0.55–0.81)	<0.001
Rehospitalization for HF	22.5%	16.1%	0.69 (0.58–0.81)	<0.001
Stroke	2.1%	1.8%	0.90 (0.62–1.33)	0.614
PPI	18.5%	16.8%	0.91 (0.77–1.09)	0.307
MI	2.1%	1.8%	0.84 (0.50–1.41)	0.501

Abbreviations: CI, confidence interval; HF, heart failure; HR, hazard ratio; MI, myocardial infarction; PPI, permanent pacemaker implantation; SHR, sub-distribution hazard ratio.

## Data Availability

The data are not publicly available due to ethical and privacy policy reasons.

## Supplementary Materials

The following supporting information can be downloaded at: https://www.mdpi.com/article/10.3390/jcm11051164/s1, Supplementary File S1: statistics details, supplementary analyses, tables and figures; Figure S1: Balance of standardized mean differences among baseline variables before and after adjustment; Figure S2: One-year Kaplan-Meier survival curve and landmark analysis for all-cause death in the transfemoral cohort after inverse propensity of treatment weighting adjustment; Figure S3: One-year Fine and Gray cumulative incidence analysis for rehospitalization for heart failure and stroke in the transfemoral cohort after inverse propensity of treatment weighting adjustment; Figure S4: One-year Kaplan-Meier survival curves for all-cause death in patients receiving SA-PIEN family or CoreValve/Evolut family devices after independent inverse propensity of treatment weighting adjustment; Table S1: Procedural characteristics after inverse probability of treatment weighting (IPTW) and propensity score matching (PSM) adjustment; Table S2: In-hospital outcomes after inverse probability of treatment weighting (IPTW) and propensity score matching (PSM) adjustment; Table S3: Landmark analyses for all-cause death after inverse probability of treatment weighting (IPTW) and propensity score matching (PSM) adjustment; Table S4: One-year outcomes after inverse probability of treatment weighting (IPTW) and propensity score matching (PSM) adjustment in the transfemoral approach cohort; Table S5: Landmark analyses for all-cause death after inverse probability of treatment weighting (IPTW) and propensity score matching (PSM) adjustment in the transfemoral approach cohort; Table S6: One-year all-cause death in TF-TAVI patients receiving SAPIEN family or CoreValve/Evolut family transcatheter aortic valves after independent inverse propensity of treatment weighting (IPTW) adjustment; Table S7: Multivariate logistic regression analysis of procedural and post-procedural variables associated with all-cause death at 1 year; Supplementary File S2: OBSERVANT II research group.

## Appendix A. OBSERVANT II Investigators

### Appendix A.1. Coordination

Fulvia Seccareccia, Paola D’Errigo, Stefano Rosato, Gabriella Badoni. National Centre for Global Health—Istituto Superiore di Sanità, Rome, Italy

### Appendix A.2. Collaborators for the “Ricerca Finalizzata 2016” (PE-2016-02364619)

Corrado Tamburino (PI), Davide Capodanno (Co-PI), Marco Barbanti. A.O.U. Policlinico “G. Rodolico—San Marco”—University of Catania, Catania, Italy

Fausto Biancari. Helsinki University Hospital and University of Helsinki, Helsinki, Finland; Oulu University Hospital, Oulu, Finland

Giovanni Baglio, Francesco Cerza. Agenzia Nazionale per i Servizi Sanitari Regionali (Age.Na.S)—PNE, Rome, Italy

Andrea Marcellusi. Faculty of Economics, University of Rome “Tor Vergata”, Rome, Italy

### Appendix A.3. Representatives of the Scientific Societies

- IFC—Italian Federation of Cardiology

Gennaro Santoro. Fondazione “G. Monasterio” CNR/Tuscany Region for the Medical Research and Public Health, Massa, Italy

Gian Paolo Ussia. Campus Bio-Medico University of Rome, Rome, Italy

- GISE—Italian Society of Interventional Cardiology

Giuseppe Musumeci. S. Croce e Carle Hospital, Cuneo.

Francesco Bedogni. IRCCS Policlinico S. Donato, S. Donato Milanese, Milan, Italy

Sergio Berti. Fondazione “G. Monasterio” CNR/Tuscany Region for the Medical Research and Public Health, Massa, Italy

Giuseppe Tarantini. University of Padova, Padova, Italy

- ITACTA—Italian Association of Cardiothoracic Anesthesia Massimo Baiocchi. Policlinico Sant’Orsola, Bologna, Italy

Marco Ranucci. IRCCS Policlinico S. Donato, S. Donato Milanese, Milan, Italy

### Appendix A.4. Institutional Collaborations

- National

Domenico Mantoan. Agenzia Nazionale per i Servizi Sanitari Regionali (Age.Na.S), Rome, Italy

- Italian Regional Authorities

Rossana De Palma. Emilia Romagna Region

Salvatore Scondotto. Sicily Region

Anna Orlando. Piemonte Region.

### Appendix A.5. Participating Centers

1. A.O.U. Città della Salute e della Scienza di Torino (TO)—Mauro Rinaldi, Stefano Salizzoni.
2. A.O. S. Croce e Carle (CN)—Giuseppe Musumeci, Giorgio Baralis.
3. A.O. SS. Antonio e Biagio e Cesare Arrigo (AL)—Gianfranco Pistis, Maurizio Reale.
4. I.R.C.C.S Policlinico San Donato (San Donato Milanese—MI)—Francesco Bedogni, Giovanni Bianchi.
5. I.R.C.C.S Multimedica (Sesto San Giovanni—MI)—Flavio Airoldi, Iassen Michev.
6. Fondazione I.R.C.C.S. Policlinico San Matteo (PV)—Maurizio Ferrario, Umberto Canosi.
7. ASST Lecco—Ospedale “A. Manzoni” (LC)—Luigi Piatti, Gianluca Tiberti.
8. ASST degli Spedali Civili—Presidio Ospedaliero di Brescia (BS)—Federica Ettori (retired), Salvatore Curello, Marianna Adamo.
9. I.R.C.C.S Ospedale San Raffaele (MI)—Antonio Colombo, Matteo Montorfano, Marco Ancona.
10. ASST Monza & Brianza—Ospedale S. Gerardo (MB)—Virgilio Colombo, Ivan Calchera.
11. Fondazione Poliambulanza (BS)—Ornella Leonzi, Diego Maffeo.
12. ASST Papa Giovanni XXIII (BG)—Orazio Valsecchi, Federica Roncali, Angelina Vassileva.
13. Policlinico di Monza (MB)—Filippo Scalise, Giovanni Sorropago.
14. A.O. di Padova—Centro Gallucci (PD)—Giuseppe Tarantini, Alessandro Schiavo.
15. Hesperia Hospital (MO)—Giuseppe D’Anniballe, Davide Gabbieri.
16. A.O.U. di Parma (PR)—Luigi Vignali, Michela Bollettino.
17. A.O.U. Careggi (FI)—Carlo Di Mario, Francesco Meucci.
18. A.O.U. Senese—Ospedale Santa Maria alle Scotte (SI)—Carlo Pierli (retired), Massimo Fineschi, Alessandro Iadanza.
19. Fondazione Toscana Gabriele Monasterio—Ospedale del Cuore “G. Pasquinucci” (MS)—Sergio Berti, Giuseppa Lo Surdo.
20. Ospedale San Filippo Neri (RM)—Giulio Speciale, Andrea Bisciglia.
21. Fondazione Policlinico Universitario Agostino Gemelli IRCCS—Università Cattolica del Sacro Cuore (RM)—Carlo Trani, Diana Verdirosi.
22. A.O. San Camillo Forlanini (RM)—Roberto Violini, Laura Zappavigna.
23. A.O. San Giuseppe Moscati (AV)—Emilio Di Lorenzo, Michele Capasso.
24. A.O.U. Federico II (NA)—Giovanni Esposito, Fabio Magliulo.
25. A.O.U. OO.RR. San Giovanni di Dio e Ruggi d’Aragona (SA)—Pietro Giudice, Tiziana Attisano.
26. A.O.U.C. Policlinico di Bari (BA)—Alessandro Santo Bortone, Emanuela De Cillis.
27. A.O.U. Policlinico-Vittorio Emanuele, Università di Catania (CT)—Corrado Tamburino, Marco Barbanti.
28. Centro Cuore Morgagni—Pedara (CT)—Sebastiano Immè, Martina Patanè.

## References

[1] Popma J.J., Michael Deeb G., Yakubov S.J., Mumtaz M., Gada H., O’Hair D., Bajwa T., Heiser J.C., Merhi W., Kleiman N.S. (2019). Transcatheter aortic-valve replacement with a self-expanding valve in low-risk patients. N. Engl. J. Med..

[2] Mack M.J., Leon M.B., Thourani V.H., Makkar R., Kodali S.K., Russo M., Kapadia S.R., Malaisrie S.C., Cohen D.J., Pibarot P. (2019). Transcatheter Aortic-Valve Replacement with a Balloon-Expandable Valve in Low-Risk Patients. N. Engl. J. Med..

[3] Barbanti M., Costa G. (2021). Highlights from the 2020 ACC/AHA guidelines on valvular heart disease. EuroIntervention.

[4] Barbanti M., Buccheri S., Rodés-Cabau J., Gulino S., Généreux P., Pilato G., Dvir D., Picci A., Costa G., Tamburino C. (2017). Transcatheter aortic valve replacement with new-generation devices: A systematic review and meta-analysis. Int. J. Cardiol..

[5] Auffret V., Lefevre T., Van Belle E., Eltchaninoff H., Iung B., Koning R., Motreff P., Leprince P., Verhoye J.P., Manigold T. (2017). Temporal Trends in Transcatheter Aortic Valve Replacement in France: FRANCE 2 to FRANCE TAVI. J. Am. Coll. Cardiol..

[6] Carroll J.D., Mack M.J., Vemulapalli S., Herrmann H.C., Gleason T.G., Hanzel G., Deeb G.M., Thourani V.H., Cohen D.J., Desai N. (2020). STS-ACC TVT Registry of Transcatheter Aortic Valve Replacement. J. Am. Coll. Cardiol..

[7] Tamburino C., Barbanti M., D’Errigo P., Ranucci M., Onorati F., Covello R.D., Santini F., Rosato S., Santoro G., Fusco D. (2015). 1-Year Outcomes After Transfemoral Transcatheter or Surgical Aortic Valve Replacement. J. Am. Coll. Cardiol..

[8] Seccareccia F., Tarantini G., Bedogni F., Berti S., Santoro G., Tamburino C., Ussia G.P., Barbanti M., Baiocchi M., Ranucci M. (2017). OBSERVANT II: OBservational Study of Effectiveness of transcatheter aortic valve implantation with new geneRation deVices for severe Aortic steNosis Treatment. Study protocol. G. Ital. Cardiol..

[9] Spence M.S., Baan J., Iacovelli F., Martinelli G.L., Muir D.F., Saia F., Bortone A.S., Densem C.G., Owens C.G., van der Kley F. (2020). Prespecified Risk Criteria Facilitate Adequate Discharge and Long-Term Outcomes After Transfemoral Transcatheter Aortic Valve Implantation. J. Am. Heart Assoc..

[10] Costa G., Barbanti M., Picci A., Todaro D., La Spina K., Di Simone E., D’Arrigo P., Criscione E., Valvo R., Reddavid C. (2020). Predictors and Safety of Next-Day Discharge in Unselected Patients Undergoing Transfemoral Transcatheter Aortic Valve Implantation. EuroIntervention.

[11] Barbanti M., Gulino S., Costa G., Tamburino C. (2018). Optimization and simplification of transcatheter aortic valve implantation therapy. Expert Rev. Cardiovasc. Ther..

[12] Barbanti M., van Mourik M.S., Spence M.S., Icovelli F., Martinelli G.L., Muir D.F., Saia F., Bortone A.S., Densem C.G., van der Kley F. (2019). Optimising patient discharge management after transfemoral transcatheter aortic valve implantation: The multicentre European FAST-TAVI trial. EuroIntervention.

[13] Wood D.A., Lauck S.B., Cairns J.A., Humphries K.H., Cook R., Welsh R., Leipsic J., Genereux P., Moss R., Jue J. (2019). The Vancouver 3M (Multidisciplinary, Multimodality, But Minimalist) Clinical Pathway Facilitates Safe Next-Day Discharge Home at Low-, Medium-, and High-Volume Transfemoral Transcatheter Aortic Valve Replacement Centers. JACC Cardiovasc. Interv..

