# Supplementary material for: One-Year Outcomes and Trends over Two Eras of Transcatheter Aortic Valve Implantation in Real-World Practice
Source: J Clin Med. 2022 Feb 22;11(5):1164. doi: 10.3390/jcm11051164 (PMC8911125; doi:10.3390/jcm11051164)
Supplement: Supplementary file 1 [file jcm-11-01164-s001.zip › jcm-1540717-supplementary.pdf]

One-Year Outcomes and Trends over Two Eras of Transcatheter Aortic Valve Implantation in Real-World Practice

Supplementary File S1

Statistical analysis

Categorical variables are reported as counts and percentages. Continuous variables are reported as medians and interquartile ranges (IQRs). Continuous variables were compared with the t-test or Mann-Whitney U test for paired samples, and categorical variables were compared with the chi-square statistics, Fischer's exact or McNemar tests for paired samples as appropriate.

To account for the non-randomized design of our study, two adjustment methods based on propensity score (PS) were used. The PS was estimated using a logistic regression model according to a non-parsimonious approach. Variables included in the PS are reported in **Supplementary Figure S1.** Inverse probability of treatment weighting (IPTW) based on propensity score (PS) was used as primary tool to adjust for baseline confounding variables between comparing groups. One-to-one PS matching with the nearest neighbor method was used as sensitivity analysis. A caliper width of 0.1 x standard deviation (SD) of PS logit was used to select two paired samples with minimum imbalance in baseline characteristics. Balance between baseline characteristics was estimated using standardized mean difference (SMD) and values <0.1 were considered an acceptable balance between covariates (**Supplementary Figure S1**).

Time-to-event curve for primary outcome was constructed with the Kaplan-Meier estimates in PSM cohorts as well as adjusted by the IPTW. Hazard ratio (HR) for all-cause death was calculated using the IPTW-adjusted Cox proportional-hazard regression model. Cumulative incidence functions of stroke, MI, repeat hospitalization for HF and PPI were estimated using a competing-risk regression using Fine and Gray method adjusted by the IPTW. In these analyses, death has been considered a competing event because patients under observation might have died preventing the event of interest to occur.

Finally, independent predictors of all-cause death were assessed using IPTW-adjusted multivariable logistic regression model. All statistical tests were performed two-tailed, and a *p*-value <0.05 was considered as the threshold for statistical significance. Statistical analyses were performed using R 3.4 (R Foundation for Statistical Computing, Vienna, Austria) software equipped with "twang" package.

Results of additional analyses

A secondary analysis was conducted considering only patients undergoing transfemoral (TF) TAVI, and treated with first generation CoreValve (Medtronic Inc, Minneapolis) and SAPIEN XT (Edwards Lifesciences, Irvine, CA) devices in the OBSERVANT study (n = 1430), and with new generation Evolut R/PRO (Medtronic Inc, Minneapolis), SAPIEN 3 (Edwards Lifesciences, Irvine, CA), Engager (Edwards Lifesciences, Irvine, CA), Acurate Neo TF (Boston Scientific, Marlborough, MA), Portico (Abbott Cardiovascular, Chicago, Illinois), Lotus (Boston Scientific, Marlborough, MA) devices in the OBSERVANT II study (n = 2511).

After IPTW adjustment, OBSERVANT II patients had a lower risk of all-cause death [10.8% vs. 15.0%, HR 0.71 (95% CI 0.57–0.88); $p < 0.01$] at 1 year, compared to OBSERVANT patients (**Supplementary Table S4 and Supplementary Figure S2**).

The benefit in terms of all-cause death was mostly confined within the first 30 days [2.2% vs. 4.3%, HR 0.52 (95% CI 0.34–0.77); $p < 0.01$], being not statistically significant thereafter [8.7% vs. 11.1%, HR 0.79 (0.62–1.01); $p = 0.06$] (**Supplementary Table S5 and Supplementary Figure S2**).

The risk of rehospitalization for HF [14.6% vs. 19.4%, HR 0.73 (95% CI 0.61–0.88); $p < 0.01$] was lower for OBSERVANT II patients, whereas rates of stroke [3.4% vs. 3.7%, HR 0.92 (95% CI 0.62–1.36); $p = 0.68$], PPI [17.4% vs. 18.7%, HR 0.94 (95% CI 0.78–1.13); $p = 0.50$] and MI [1.7% vs. 1.8%, HR 0.91 (95% CI 0.54–1.53); $p = 0.71$] were similar between patients of OBSERVANT II and OBSERVANT studies at 1 year (**Supplementary Table S4 and Supplementary Figure S3**).

In a pre-specified exploratory analysis, patients undergoing TF-TAVI with first generation CoreValve or SAPIEN XT transcatheter aortic valves (TAVs) in the OBSERVANT study were compared with those treated with the respective device iterations Evolut R/PRO and SAPIEN 3 in the OBSERVANT II study.

At 1 year, no difference was observed between patients treated with SAPIEN family valves in OBSERVANT II and OBSERVANT studies in terms of all-cause death [9.7% vs. 11.9%; HR 0.80 (95% CI 0.47–1.36); $p = 0.42$], whereas patients treated with new generation Evolut R/PRO valves in the OBSERVANT II study had a lower risk of all-cause death [12.3% vs. 16.5%, HR 0.73 (95% CI 0.55–0.96); $p = 0.03$] compared to those receiving first generation CoreValve TAV in the OBSERVANT study (**Supplementary Table S6 and Supplementary Figure S4**).

Finally, in the IPTW-adjusted multivariable logistic regression analysis, undergoing TAVI in the period of OBSERVANT II study was found to be a protective factor of all-cause death [0.79 (95% CI 0.60–1.05); $p = 0.02$] (**Supplementary Table S7)**.

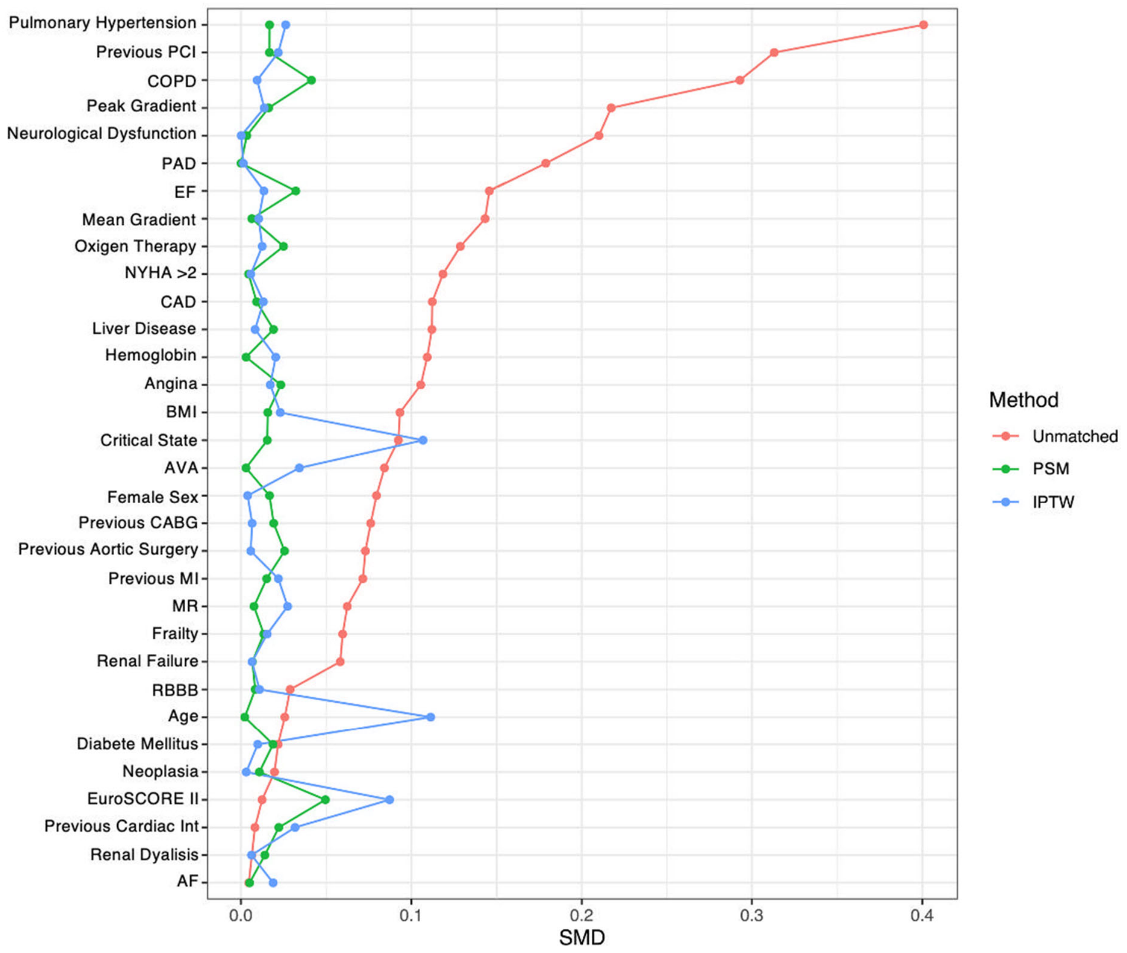

Figure S1. Balance of standardized mean differences among baseline variables before and after adjustment.

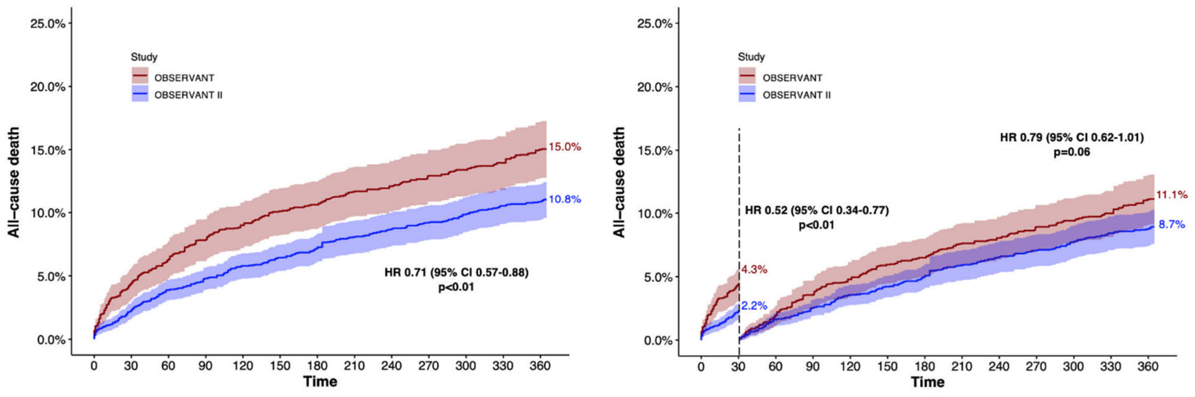

Figure S2. One-year Kaplan-Meier survival curve and landmark analysis for all-cause death in the transfemoral cohort after inverse propensity of treatment weighting adjustment.

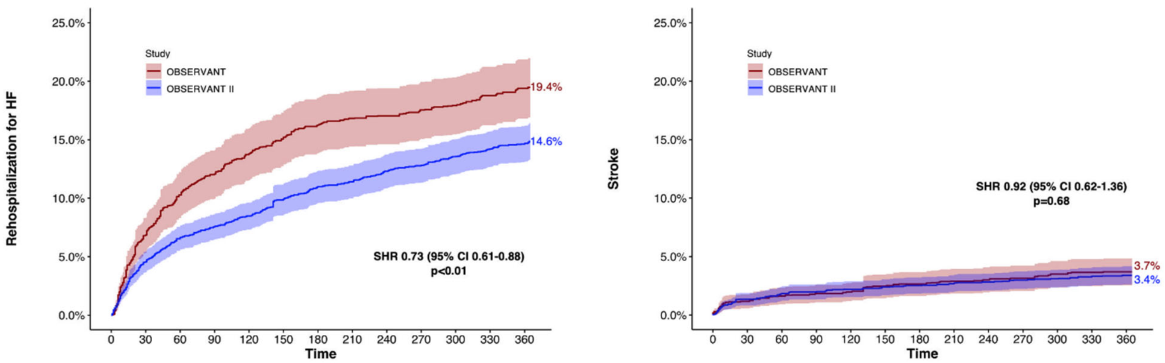

Figure S3. One-year Fine and Gray cumulative incidence analysis for rehospitalization for heart failure and stroke in the transfemoral cohort after inverse propensity of treatment weighting adjustment.

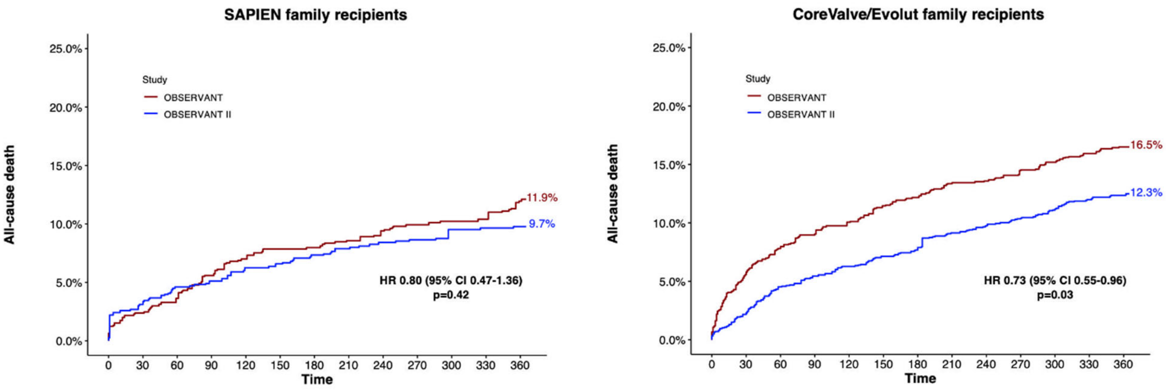

Figure S4. One-year Kaplan-Meier survival curves for all-cause death in patients receiving SAPIEN family or CoreValve/Evolut family devices after independent inverse propensity of treatment weighting adjustment.

Table S1. Procedural characteristics after inverse probability of treatment weighting (IPTW) and propensity score matching (PSM) adjustment.

	**IPTW adjustment**			**PSM adjustment**		
	**OBS (n = 1811)**	**OBS II (n = 2939)**	***p*-value**	**OBS (n = 1451)**	**OBS II (n = 1451)**	***p*-value**
Concomitant PCI, n (%)	69 (3.8)	170 (5.8)	0.022	46 (3.2)	74 (5.1)	0.012
General Anesthesia, n (%)	661 (36.5)	535 (18.2)	<0.01	514 (35.4)	257 (17.7)	<0.01
Approach, n (%)			<0.01			<0.01
Transfemoral	1492 (82.4)	2672 (90.9)		1193 (82.2)	1318 (90.8)	
Transapical	243 (13.4)	126 (4.3)		195 (13.4)	65 (4.5)	
Transaxillary	58 (3.2)	120 (4.1)		51 (3.5)	59 (4.1)	
Others	16 (0.9)	24 (0.8)		12 (0.8)	9 (0.6)	
Devices, n (%)			<0.01			<0.01
Not available	65 (3.6)	9 (0.3)		57 (3.9)	3 (0.2)	
Sapien XT	842 (46.5)	141 (4.8)		676 (46.6)	72 (5.0)	
CoreValve	904 (49.9)	44 (1.5)		718 (49.5)	17 (1.2)	
Sapien 3	0.0 (0.0)	779 (26.5)		0 (0.0)	376 (25.9)	
Evolut R	0.0 (0.0)	1137 (38.7)		0 (0.0)	559 (38.5)	
Evolut PRO	0.0 (0.0)	335 (11.4)		0 (0.0)	175 (12.1)	
Acurate Neo	0.0 (0.0)	288 (9.8)		0 (0.0)	144 (9.9)	
Portico	0.0 (0.0)	188 (6.4)		0 (0.0)	97 (6.7)	
Lotus	0.0 (0.0)	12 (0.4)		0 (0.0)	4 (0.3)	
Engage	0.0 (0.0)	6 (0.2)		0 (0.0)	4 (0.3)	

Abbreviations: IPTW, Iinverse probability of treatment weighting; PCI, percutaneous coronary intervention; PSM, propensity score matching.

Table S2. In-hospital outcomes after inverse probability of treatment weighting (IPTW) and propensity score matching (PSM) adjustment.

	IPTW adjustment			PSM adjustment		
	**OBS (n = 1811)**	**OBS II (n = 2939)**	***p*-value**	**OBS (n = 1451)**	**OBS II (n = 1451)**	***p*-value**
Prosthesis migration, n (%)	24 (1.3)	41 (1.4)	0.937	22 (1.5)	24 (1.7)	0.882
Myocardial infarction, n (%)	16 (0.9)	9 (0.3)	0.011	13 (0.9)	3 (0.2)	0.021
Tamponade, n (%)	81 (4.5)	29 (1.0)	<0.01	70 (4.8)	14 (1.0)	<0.01
Vascular injury, n (%)	114 (6.3)	71 (2.4)	<0.001	90 (6.2)	35 (2.4)	<0.001
Complication of left ventricular apex, n (%)	14 (0.8)	3 (0.1)	<0.001	13 (0.9)	2 (0.1)	0.007
Permanent pacemaker implantation, (%)	270 (14.9)	388 (13.2)	0.142	221 (15.2)	189 (13.0)	0.098
Conversion to open surgery, n (%)	7 (0.4)	9 (0.3)	0.316	9 (0.6)	4 (0.3)	0.266
Stroke, n (%)	20 (1.1)	18 (0.6)	0.075	18 (1.2)	8 (0.6)	0.074
Shock, n (%)	49 (2.7)	38 (1.3)	0.006	42 (2.9)	20 (1.4)	0.007
Blood transfusion, n (%)	587 (32.4)	514 (17.5)	<0.001	465 (32.0)	278 (19.2)	<0.001
Acute kidney injury, n (%)	120 (6.6)	41 (1.4)	<0.001	71 (4.9)	22 (1.5)	<0.001
Infection, n (%)	107 (5.9)	144 (4.9)	0.205	88 (6.1)	79 (5.4)	0.524
Paravalvular regurgitation, n (%)			0.013			0.027
None/trivial	933 (51.5)	1614 (54.9)		748 (51.6)	806 (55.5)	
Mild	688 (38.0)	1102 (37.5)		551 (38.0)	529 (36.5)	
Moderate	177 (9.8)	215 (7.3)		141 (9.7)	112 (7.7)	
Severe	13 (0.7)	9 (0.3)		11 (0.8)	4 (0.3)	
Aortic valve peak gradient, median (IQR)	19.0 (13.0–22.0)	15.0 (10.8–19.0)	<0.001	19.0 (13.0–21.5)	15.0 (11.0–20.0)	<0.001
Aortic valve mean gradient, median (IQR)	10.0 (7.0–12.0)	8.0 (5.0–11.0)	<0.001	10.0 (7.0–11.0)	8.0 (5.0–11.0)	<0.001
ICU length of stay, median (IQR)	2.00 (1.00, 3.00)	1.0 (0.0–2.0)	<0.001	2.0 (1.0–3.0)	1.0 (0.0–2.0)	<0.001
Monitored ward length of stay, median (IQR)	0.00 (0.0–3.0)	1.0 (0.0–3.0)	<0.001	0.0 (0.0–3.0)	1.0 (0.0–3.0)	<0.001
Hospital length of stay, median (IQR)	11.0 (8.0–17.0)	9.0 (6.0–14.0)	<0.001	10.0 (8.0–16.0)	9.0 (6.0–14.0)	<0.001

Abbreviations: IPTW, inverse probability of treatment weighting; PSM, propensity score matching.

Table S3. Landmark analyses for all-cause death after inverse probability of treatment weighting (IPTW) and propensity score matching (PSM) adjustment.

	OBS	OBS II	HR (95%CI)	*p*-value
**IPTW adjustment**	**N= 1811**	**N= 2939**		
From 0 to 30 days	5.0%	2.2%	0.45 (0.30–0.65)	<0.01
From 30 days to 1 year	11.8%	8.5%	0.71 (0.57–0.88)	<0.01
**PSM adjustment**	**N= 1451**	**N= 1451**		
From 0 to 30 days	4.9%	2.3%	0.48 (0.32–0.72)	<0.01
From 30 days to 1 year	11.8%	8.9%	0.74 (0.59–0.94)	0.012

Abbreviations: IPTW, inverse probability of treatment weighting; PSM, propensity score matching.

Table S4. One-year outcomes after inverse probability of treatment weighting (IPTW) and propensity score matching (PSM) adjustment in the transfemoral approach cohort.

	OBS	OBS II	HR/SHR (95%CI)	*p*-value
**IPTW adjustment**	**N = 1450**	**N = 2511**		
All-cause death	15.0%	10.8%	0.71 (0.57–0.88)	0.001
Rehospitalization for HF	19.4%	14.6%	0.73 (0.61–0.88)	0.001
Stroke	3.7%	3.4%	0.92 (0.62–1.36)	0.677
PPI	18.7%	17.4%	0.94 (0.78–1.13)	0.495
MI	1.8%	1.7%	0.91 (0.54–1.53)	0.710
**PSM adjustment**	**N = 1163**	**N = 1163**		
All-cause death	14.7%	11.3%	0.76 (0.61–0.95)	0.017
Rehospitalization for HF	19.9%	15.1%	0.74 (0.61–0.89)	0.002
Stroke	4.0%	3.4%	0.85 (0.55–1.30)	0.446
PPI	18.8%	18.6%	1.00 (0.83–1.21)	0.961
MI	1.7%	1.8%	1.05 (0.57–1.94)	0.878

Abbreviations: CI, confidence interval; HF, heart failure; HR, hazard ratio; MI, myocardial infarction; PPI, permanent pacemaker implantation; PSM, propensity score matching; SHR, sub-distribution hazard ratio.

Table S5. Landmark analyses for all-cause death after inverse probability of treatment weighting (IPTW) and propensity score matching (PSM) adjustment in the transfemoral approach cohort.

	OBS	OBS II	HR (95%CI)	*p*-value
**IPTW adjustment**	**N = 1450**	**N = 2511**		
From 0 to 30 days	4.3%	2.2%	0.52 (0.34–0.77)	0.001
From 30 days to 1 year	11.1%	8.7%	0.79 (0.62–1.01)	0.063
**PSM adjustment**	**N = 1163**	**N = 1163**		
From 0 to 30 days	4.1%	2.4%	0.59 (0.38–0.94)	0.024
From 30 days to 1 year	10.9%	9.0%	0.83 (0.64–1.07)	0.155

Abbreviations: IPTW, inverse probability of treatment weighting; PSM, propensity score matching.

Table S6. One-year all-cause death in TF-TAVI patients receiving SAPIEN family or CoreValve/Evolut family transcatheter aortic valves after independent inverse propensity of treatment weighting (IPTW) adjustment.

	OBS	OBS II	HR (95%CI)	*p*-value
SAPIEN family TAVs recipients	11.9% (n = 598)	9.7% (n = 675)	0.80 (0.47–1.36)	0.418
CoreValve/Evolut family TAVs recipients	16.5% (n = 832)	12.3% (n = 1352)	0.73 (0.55–0.96)	0.026

Abbreviations: HR, hazard ratio; TAVs, transcatheter aortic valves.

Table S7. Multivariate logistic regression analysis of procedural and post-procedural variables associated with all-cause death at 1 year.

	**Odds Ratio**	***p*-value**
OBSERVANT II study	0.76 (0.64–0.92)	0.016
Transfemoral approach	0.79 (0.60–1.05)	0.304
Moderate-to-severe PVR	1.40 (1.06–1.83)	0.041
Permanent pacemaker implantation	1.21 (0.94–1.53)	0.204
Acute kidney injury	3.93 (2.83–5.44)	<0.001
Vascular injury	1.47 (1.00–2.11)	0.107
Stroke	11.75 (6.13–23.43)	<0.001
Myocardial infarction	7.51 (3.41–16.96)	<0.001
Device Migration	1.08 (0.51–2.10)	0.857
Complication of left ventricular apex	1.32 (0.42–3.74)	0.619
Coronary Artery Disease	1.16 (0.78–1.69)	0.542
General anesthesia	1.02 (0.81–1.28)	0.909
Conversion to surgery	21.35 (6.48–97.74)	<0.001
Infection	1.57 (1.12–2.16)	0.022
Blood transfusion	1.23 (1.00–1.50)	0.141

Abbreviations: PVR: paravalvular regurgitation.

OBSERVANT II RESEARCH GROUP

Coordination

Fulvia Seccareccia, Paola D'Errigo, Stefano Rosato, Gabriella Badoni. National Centre for Global Health - Istituto Superiore di Sanità, Rome, Italy;

Collaborators for the "Ricerca Finalizzata 2016" (PE-2016-02364619)

Corrado Tamburino (PI), Davide Capodanno (Co-PI), Marco Barbanti. A.O.U. Policlinico "G. Rodolico – San Marco" – University of Catania, Catania, Italy
Fausto Biancari. Helsinki University Hospital and University of Helsinki, Helsinki, Finland; Oulu University Hospital, Oulu, Finland
Giovanni Baglio, Francesco Cerza. Agenzia Nazionale per i Servizi Sanitari Regionali (Age.Na.S) – PNE, Rome, Italy
Andrea Marcellusi. Faculty of Economics, University of Rome "Tor Vergata", Rome, Italy

Representatives of the Scientific Societies

- IFC - Italian Federation of Cardiology

Gennaro Santoro. Fondazione "G. Monasterio" CNR/Tuscany Region for the Medical Research and Public Health, Massa, Italy
Gian Paolo Ussia. Campus Bio-Medico University of Rome, Rome, Italy

- GISE – Italian Society of Interventional Cardiology

Giuseppe Musumeci. S. Croce e Carle Hospital, Cuneo
Francesco Bedogni. IRCCS Policlinico S. Donato, S. Donato Milanese, Milan, Italy
Sergio Berti. Fondazione "G. Monasterio" CNR/Tuscany Region for the Medical Research and Public Health, Massa, Italy
Giuseppe Tarantini. University of Padova, Padova, Italy

- ITACTA - Italian Association of Cardiothoracic Anesthesia

Massimo Baiocchi. Policlinico Sant'Orsola, Bologna, Italy
Marco Ranucci. IRCCS Policlinico S. Donato, S. Donato Milanese, Milan, Italy

Institutional collaborations

- National

Domenico Mantoan. Agenzia Nazionale per i Servizi Sanitari Regionali (Age.Na.S), Rome, Italy

- Italian Regional Authorities

Rossana De Palma. Emilia Romagna Region
Salvatore Scondotto. Sicily Region
Anna Orlando. Piemonte Region

Participating hemodynamic centers

1. A.O.U. Città della Salute e della Scienza di Torino (TO) - Mauro Rinaldi, Stefano Salizzoni
2. A.O. S. Croce e Carle (CN) - Giuseppe Musumeci, Giorgio Baralis
3. A.O. SS. Antonio e Biagio e Cesare Arrigo (AL) - Gianfranco Pistis, Maurizio Reale
4. I.R.C.C.S Policlinico San Donato (San Donato Milanese - MI) - Francesco Bedogni, Giovanni Bianchi
5. I.R.C.C.S Multimedica (Sesto San Giovanni - MI) - Flavio Airoldi, Iassen Michev
6. Fondazione I.R.C.C.S. Policlinico San Matteo (PV) - Maurizio Ferrario, Umberto Canosi
7. ASST Lecco - Ospedale "A. Manzoni" (LC) - Luigi Piatti, Gianluca Tiberti
8. ASST degli Spedali Civili - Presidio Ospedaliero di Brescia (BS) - Federica Ettori (retired), Salvatore Curello, Marianna Adamo
9. I.R.C.C.S Ospedale San Raffaele (MI) - Antonio Colombo, Matteo Montorfano, Marco Ancona,
10. ASST Monza & Brianza - Ospedale S. Gerardo (MB) - Virgilio Colombo, Ivan Calchera
11. Fondazione Poliambulanza (BS) - Ornella Leonzi, Diego Maffeo
12. ASST Papa Giovanni XXIII (BG) - Orazio Valsecchi, Federica Roncali, Angelina Vassileva
13. Policlinico di Monza (MB) - Filippo Scalise, Giovanni Sorropago
14. A.O. di Padova - Centro Gallucci (PD) - Giuseppe Tarantini, Alessandro Schiavo
15. Hesperia Hospital (MO) - Giuseppe D'Anniballe, Davide Gabbieri
16. A.O.U. di Parma (PR) - Luigi Vignali, Michela Bollettino
17. A.O.U. Careggi (FI) - Carlo Di Mario, Francesco Meucci
18. A.O.U. Senese - Ospedale Santa Maria alle Scotte (SI) - Carlo Pierli (retired), Massimo Fineschi, Alessandro Iadanza
19. Fondazione Toscana Gabriele Monasterio - Ospedale del Cuore "G. Pasquinucci" (MS) - Sergio Berti, Giuseppa Lo Surdo
20. Ospedale San Filippo Neri (RM) - Giulio Speciale, Andrea Bisciglia
21. Fondazione Policlinico Universitario Agostino Gemelli IRCCS - Università Cattolica del Sacro Cuore (RM) - Carlo Trani, Diana Verdirosi
22. A.O. San Camillo Forlanini (RM) - Roberto Violini, Laura Zappavigna
23. A.O. San Giuseppe Moscati (AV) - Emilio Di Lorenzo, Michele Capasso
24. A.O.U. Federico II (NA)- Giovanni Esposito, Fabio Magliulo
25. A.O.U. OO.RR. San Giovanni di Dio e Ruggi d'Aragona (SA) - Pietro Giudice, Tiziana Attisano
26. A.O.U.C. Policlinico di Bari (BA) - Alessandro Santo Bortone, Emanuela De Cillis
27. A.O.U. Policlinico-Vittorio Emanuele, Università di Catania (CT) - Corrado Tamburino, Marco Barbanti
28. Centro Cuore Morgagni - Pedara (CT) - Sebastiano Immè, Martina Patanè

OBSERVANT I RESEARCH GROUP

The **Research Group and participating centers of the OBSERVANT I study** have been previously listed in: Barbanti M, Tamburino C, D'Errigo P, et al. Five-year outcomes of transfemoral transcatheter aortic valve replacement or surgical aortic valve replacement in a real world population. Circ Cardiovasc Interv. 2019;12:e007825 - Supplemental Material.
